# University Social Responsibility in China: The Mediating Role of Green Psychological Capital

**DOI:** 10.3390/ijerph20043634

**Published:** 2023-02-18

**Authors:** Yu-Shan Chen, Xin Yan, Chor-Beng Anthony Liew

**Affiliations:** 1 Department of Business Administration, National Taipei University, 151 University Rd., San Shia District, New Taipei City 237303, Taiwan; 2 School of Finance and Accounting, Fuzhou University of International Studies and Trade, 28 Yuhuan Rd., Changle District, Fuzhou 350202, China

**Keywords:** university social responsibility (USR), generation Z, green shared vision, green psychological capital, organizational citizenship behavior for the environment (OCBE)

## Abstract

Generation Z represents the young people of today. They are considered as “digitally literate” and were born between mid-to-late 1990s to early 2000s. Generation Z pays more attention to popular environmental issues such as global warming, high energy consumption, overgrazing, and university social responsibility (USR), which are present around the world. We formed a double moderated mediation exam from 910 college students in southeast China, used a new notion “green psychological capital”, and proposed it as a vital mediator. In addition, we found that green organizational ambidexterity and environmental attitude are both boundary conditions in the green shared vision organizational citizenship behavior for the environment (OCBE) link. These findings have unlocked a deeper insight into Generation Z’s green conception and offered a more comprehensive investigation on USR research. Furthermore, the amazing findings can provide a worldwide blueprint for USR studies in the long term.

## 1. Introduction

The old Chinese saying “harmony between human and nature” is present in today’s college student lives through adoption of the university social responsibility (USR) concept. The emergence of USR has integrated the environmental concept into current higher education systems, as well as promoted cooperation between universities and different social groups [1]. The perception of USR goes far beyond the practices of CSR on campus, it also means a huge reform in sustainable development to meet the challenge of the new era. 

The critical role of university social responsibility is undoubted, nevertheless there is a paucity of research about Generation Z’s OCBE in a USR context. Generation Z, who were born from the mid-to-late 1990s to the early 2000s, were gradually entering into university at the time of the study. Compared with Generation X and Generation Y, most of Generation Z are goal-oriented, well-educated, and adventurous. Generation Z are considered as “digitally literate”, representing the young people of the World Wide Web. As environmental education in China has grown in importance, a greater understanding of Generation Z’s green shared vision, psychological capital, and OCBE in USR field has become greatly significant.

China has the largest number of college students in the world with over 40 million college students aged 18–22 years. It is critical to understand the green conception among Generation Z in China, as well as their green shared vision, green attitude, and green psychological capital while being in trouble. Traditional Chinese ethics of “harmony between the heavens and humans” is relevant in modern economic construction. Therefore, research on USR is essential to discuss the green perception of China’s Generation Z.

Consequently, we seek new insight of the linkage between green shared vision and OCBE of China’s Generation Z. Even though there is a growing research interest in green shared vision and OCBE reactively [2,3,4], it may not hide the paucity of systematic studies on the relationship between green shared vision and OCBE. Moreover, several scholars have revealed green organizational ambidexterity and environmental attitude more or less affect green innovation performance [3,5], these mixed findings suggest that the green shared vision–OCBE link is far more complicated than that seen existing work. The aims of this study are to broaden the existing research and to explore the “green shared vision–OCBE” link in the USR context.

According to the aims of this study, several research questions are raised as follows:

1. Does green psychological capital really act as a mediator in the green shared vision–OCBE link?
2. How do the interactions influence the relationship between green shared vision and OCBE via green psychological capital?

**Figure 1:**
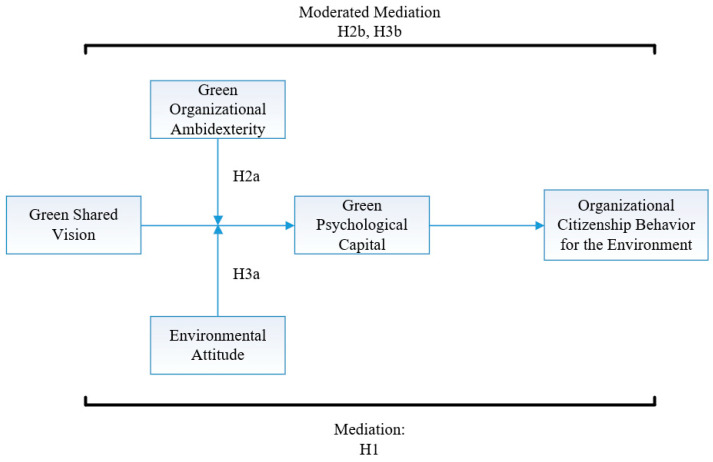
Theoretical model.

Based on social cognitive theory, positive psychology, and organizational ambidexterity, this study answered questions by making a moderated mediation exam to view the current practices of USR in southeast China. The research objective of our study is three-fold: First, we formed a moderated mediation exam to illustrate the sophisticated framework among green shared vision, green psychological capital, OCBE, green organizational ambidexterity, and environmental attitude. Second, we used a new construct, green psychological capital [6], and proposed it as a vital factor in the green shared vision–OCBE link through which Generation Z in China may be more optimistic in environmental trouble, thereby enhancing the green shared vision–OCBE relationship. Third, we argue that the indirect relationship between green shared vision and OCBE via green psychological capital become stronger when universities in China have green organizational ambidexterity and environmental attitude, as illustrated in Figure 1.

In summary, this study makes five critical contributions:

Firstly, our study echoes the prior research of USR [7]. We address the critical contribution by performing a moderated mediation test in southeast China, since this study enriches the existing research of USR by linking it into Generation Z’s environmental daily life. This empirical research is valuable and creative.

Secondly, there is a lack of systematic and empirical psychological capital analysis in environmental literature. As psychological capital has been widely used in prior research, there is a paucity of psychological capital research in environmental literature. Based on the concept of positive psychology, we expand the literature of psychological capital by using a new notion “green psychological capital” and examine the antecedents and consequences of this new notion in order to solve environmental issues in the USR context.

Thirdly, this study asserts the significance of a reasonably good fit among green shared vision, green psychological capital, OCBE, green organizational ambidexterity, and environmental attitude, so analyze the social cognitive theory and provide a more comprehensive understanding of how one’s green shared vision affects green psychological capital, which leads to a better OCBE on campus. Moreover, our study also takes the boundary conditions of green organizational ambidexterity and environmental attitude into account and proposes a model which reveals this unpacked link for the first time and adds new insight in social cognitive theory.

Finally, this study extends the organizational ambidexterity view by offering a unique but more comprehensive perspective of green organizational ambidexterity. This study asserts that green organizational ambidexterity is more than an antecedent, mediator, or consequence role [3]. In particular, we highlight the conditional influence of green organizational ambidexterity, and thus advance the organizational ambidexterity view and contributed to the environmental management literature in USR context.

## 2. Theoretical Foundation and Research Hypotheses

### 2.1. Literature Review and Theoretical Development

In this study, four theoretical perspectives are used as building blocks of explaining USR: social cognitive theory, positive psychology, broaden-and-build theory, and organizational ambidexterity. Green shared vision and green psychological capital, as well as green organizational ambidexterity and environmental attitude, are considered as surrounding influences that conceptualize Chinese students’ OCBE. The application of social cognitive theory asserts that one’s green shared vision affects their green psychological capital, which leads to a better OCBE on the campus. This mediation relationship is also moderated by green organizational ambidexterity and environmental attitude. Nonetheless, few empirical attempts have integrated these views in a USR context.

Specifically, we apply a new concept “green psychological capital”, to extend positive psychology and the broaden-and-build theory. Positive psychology was introduced by Martin Seligman in 1998, it focuses on the importance of happiness, well-being, and “the good life”. The concept of positive psychology has been applied in numerous psychological capital studies. For example, positive psychology might also greatly influence individual sales performance and life satisfaction [8]. Furthermore, previous research on the broaden-and-build theory indicated that positive emotions have special effects which can broaden one’s exploratory thinking and various resources instantaneously, leading to positive behaviors and creativity [9]. Lyngdoh et al. [10] suggested that individuals with more positive emotions are more joyful and tend to have a stronger pressure resistance and well-being. Applying the above-mentioned findings under a USR context, we suggest that green psychological capital is a critical internal factor under which green shared vision can turn into a better OCBE.

In the first use of the construct “ambidextrous”, Duncan [11] argued that companies should optimize structures to encourage and promote innovation and creativity. Tushman and O’Reilly [12] argued that companies should explore and exploit in fierce competition. Organizational ambidexterity has been recognized as a useful theory in a great number of studies to explain the way people dealing with two contradictory things in their daily life. In this study, we consider green organizational ambidexterity as a boundary condition under which the indirect relationship between green shared vision on OCBE via green psychological capital is enhanced. Existing literature has proposed several alternatives to balance the contradictions of exploration and exploitation, but there is a paucity of relevant research in the field of environmental management.

### 2.2. Green Shared Vision and OCBE: Mediation Effects of Green Psychological Capital

Shared vision is defined as the ambitious goals of a company which guide employees for the business vision [13] as well as the combination of corporate mission and key values in an organization [14]. A shared vision provides guidance and positive environmental strategies for companies, it is vital to the development of the organization by indicating the overall purpose and direction of an organization [15]. Based on the definition by Chen et al. [4], green shared vision is considered as “a clear and common strategic direction of collective environmental goals and aspirations that has been internalized by members of an organization”.

Organizational citizenship behavior for the environment (OCBE), is defined by Boiral and Paillé [16] as the collection of eco-helping, eco-civic engagement, and eco-initiatives. Daily et al. [17] considered it as “employees’ discretionary pro-environmental acts in the organization not driven by rewards or requirements”. Paillé and Raineri [18] indicated that companies with stronger environmental policy support have higher OCBE. Moreover, OCBE can not only take the managerial engagement and organizational sustainability into account [19,20], but also create spiritual leadership [21]. Thus, OCBE has become increasingly important in various fields [22,23,24].

An individual’s psychological capital shows one’s positive mental status in life. It is a vital physical and mental resource and a critical indicator of personal well-being [25]. Generally speaking, psychological capital includes four sides, namely self-efficacy, hope, resilience, and optimism, it has been widely used in the research of satisfaction, performance, or well-being. For instance, scholars have found that psychological capital has a great effect on students’ achievement or performance [26], engagement in academic activities [27], and learning capacity [28].

Referring to psychological capital research [29], we apply a new construct, “green psychological capital”. Chen and Yan [6] defined it as “an individual’s positive psychological state during environmental activities”. There are four constructs in green psychological capital: “green self-efficacy”, “green optimism”, “green hope”, and “green resilience”.

Although few studies have tested the relationship between green shared vision and OCBE, the empirical study concerning the mediating role of green psychological capital between them is limited. Theoretically, it is critical to discuss whether the relationship between green shared vision and OCBE is mediated by green psychological capital. In the following, we provide three theoretical explanations for why green shared vision can elevate Generation Z’s green psychological capital and positive motivation, which may contribute to OCBE.

Firstly, according to social cognitive theory, one’s behavior might be influenced by the ternary interaction of dynamic environmental factors, others’ actions, and personal experiences. A shared vision can stimulate employees’ interests and necessary behaviors to achieve the organization’s goals. An elaborate and widely shared vision would influence employees’ behaviors by motivating them to common goals. Furthermore, previous research has suggested that a clear and ambitious green shared vision may contribute to improvements for employees’ OCBE [30]. Green shared vision has also been found to influence various performance and outputs of an organization, including green innovation performance of enterprises [3], product development performance [31], and employee’s environmental behavior [32].

Secondly, the mediating role of green psychological capital should be further explored in terms of positive psychology and the broaden-and-build theory. Green psychological capital includes four aspects: “green self-efficacy”, “green optimism”, “green hope”, and “green resilience”, these aspects indicate that Generation Z should keep a positive mental state when engaged in environmental activities. Emerging research has recognized the impact of psychological capital on OCB [33,34]. However, it seems to fall short in an environmental context.

Finally, it requires systematic and further studies on the relationship between green shared vision and green psychological capital in environmental field. There is a rich body of research studies that has examined the relationship between separated aspects of psychological capital (self-efficacy, optimism, hope, and resilience) and green shared vision. For example, Chen et al. [4] argued that green shared vision might motivate people’s self-efficacy by setting clear goals and Parkhill et al. [35] assert that shared vision can promote social resilience and action by providing group members with feasible actions [35]. A clear and shared vision, which can empower employees with a definite common purpose, might enhance organization resilience [36]. Moreover, a shared vision can be established through shared leadership, which is closely related to academic optimism [37]. Nevertheless, there is a paucity of systematic and empirical research on the relationship between green shared vision and green psychological capital in environmental literature.

From this standpoint, it is clear that green psychological capital performs an important role in incorporating green shared vision into Generation Z’s OCBE. Universities with green shared visions can provide green perception and environmental responsibility for students which ultimately improve the campus’s environmental outcomes.

Given the above theoretical explanations, we propose the following hypothesis.

**Hypothesis 1 (H_1_):**  *The positive relationship between green shared vision and OCBE is mediated by green psychological capital.*

### 2.3. The Moderated Mediation Effects of Green Organizational Ambidexterity

Lubatkin et al. [38] defined organizational ambidexterity as an integrated capability of an organization which positively relates to the organization’s orientation of exploration and exploitation. Jansen et al. [39] indicated that an ambidextrous organization can simultaneously explore new opportunities as well as exploit existing competencies, instead of making trade-offs between the two. Prior research on organizational ambidexterity also focuses on its relationship with entrepreneurial orientation [40], organization citizenship behavior [41], as well as economic performance [42].

Chen et al. [3] further developed the idea of green organizational ambidexterity, and defined it as “the capability for an organization to integrate and reconcile both exploratory and exploitative environmental activities”. An organization with higher green organizational ambidexterity is able to tap into short-term opportunities by its exploitation capability and achieve advantages from long-term innovation through its exploration capability. Both exploitation and exploration capabilities can be directly linked to sustainability from various perspectives [43]. The main idea of organizational ambidexterity is especially beneficial to sustainable practices [44]. Moreover, the capabilities to plan, formulate, and implement paradoxical strategies are extremely important for an organization to achieve sustainable goals [45].

We argue that green organizational ambidexterity may enhance the indirect relationship of green shared vision on OCBE via green psychological capital. That is, universities with a higher capability of green organizational ambidexterity can set clearer goals and strategic directions, thus promoting students to pay more attention to environmental activities on campus as well as cope better with difficulties and challenges during participation. To be more specific, universities with a higher capability of green organizational ambidexterity may have a more positive outlook and broader green shared vision, leading to stronger green psychological capital and better OCBE for their students. On the contrary, universities with a lower capability of green organizational ambidexterity are less likely to have a clear and definite green shared vision, and students in those universities might find it difficult to cope with difficulties and to maintain a positive outlook under stressful environmental situations, which can harm the students’ green psychological capital, environmental performance, and OCBE.

Although most of previous research only focuses on organizational ambidexterity’s antecedents and consequences, or its mediating effect, no research examines the moderation effect of green organizational ambidexterity. Chen et al. [3] demonstrated that green organizational ambidexterity significantly affects green innovation performance. Similarly, Zhao et al. [46] asserted that enterprises with higher organizational ambidexterity can better integrate resources with their suppliers, and thereby improve their environmental performance. The positive relationship between a firm’s ambidexterity and its performance has been employed in different management areas [44,47,48]. Therefore, we propose that green organizational ambidexterity can be applied in the USR context.

Given the above theoretical explanations, we propose the following hypothesis.

**Hypothesis 2 (H_2_):**  *The relationship between green shared vision and green psychological capital is moderated by green organizational ambidexterity (H_2a_). In addition, the indirect relationship between green shared vision and OCBE via green psychological capital is moderated by green organizational ambidexterity such that the indirect relationship becomes stronger as green organizational ambidexterity is higher (H_2b_).*

### 2.4. The Moderated Mediation Effects of Environmental Attitude

Drawing on social cognitive theory, one’s behavior may be affected by the ternary interaction of dynamic environmental factors, others’ actions, and attitude, and personal experience. Tan [49] defined environmental attitude as “the psychological tendency of environmental perceptions or beliefs”. Moreover, environmental attitude can be divided into two aspects, inward attitude and outward attitude; positive environmental attitude can positively turn into effective environmental protection behavior [50]. In addition, the prior literature demonstrates that environmental attitude positively relates to environmental knowledge [51], the health of ecosystem [52], and environmental purchase behaviors [53]. 

The critical role of environmental attitude has been widely explored in previous research. Tarrant and Cordell [54] posit that environmental attitude is a powerful antecedent of ecological behavior, and children develop environmental attitude and knowledge which shape their environmental values in their future lives [55]. Moreover, Zuo and Zhao [45] demonstrated the importance of environmental attitude in promoting green behaviors. Students’ environmental attitude becomes more positive after systematically learning scientific environmental protection knowledge [56]. In addition, Kaiser et al. [5] enriched the research of environmental attitude by extending the sense of personal environmental responsibility to the morality field.

However, no research combines environmental attitude with green shared vision, green psychological capital, and OCBE. Moreover, no previous literature examines the boundary condition of environmental attitude in the area of management. Therefore, we focus on environmental attitude as an intrinsic factor that can influence the indirect relationship between green shared vision and OCBE via green psychological capital.

Many studies find that work attitude is closely linked with psychological capital [57,58]. Wehrmeyer and McNeil [59] indicate that college students with better active environmental attitude and knowledge lead to better pro-environmental behaviors. In this study, we argue that environmental attitude might enhance the indirect relationship of green shared vision on OCBE via green psychological capital. That is, college students with higher environmental attitude may have more positive outlooks and higher green shared visions, leading to stronger green psychological capital and better OCBE. On the contrary, college students with unfriendly environmental attitudes are less likely to have clear environmental goals and higher aspirations, who face more difficulties under stressful environmental situations, which can ruin their green psychological capital and negatively affect their OCBE.

Given the above theoretical explanations, we propose the following hypothesis.

**Hypothesis 3 (H_3_):**  *The relationship between green shared vision and green psychological capital is moderated by environmental attitude (H_3a_). In addition, the indirect relationship between green shared vision and OCBE via green psychological capital is moderated by environmental attitude such that the indirect relationship becomes stronger as environmental attitude is higher (H_3b_).*

## 3. Methodology

### 3.1. Samples and Procedures

The respondents of questionnaire survey in this study are college students in southeast China. We used “snowball sampling” to distribute questionnaires from 1 January 2021 to 30 June 2021. We initially got in touch with student unions and associations on the campus. After describing the purpose of this research, we obtained their support and distributed the questionnaires among students widely. A total of 3500 self-administered questionnaires were distributed in 89 classes. The students from different majors took part in this survey. Before the investigation, we clearly assured the confidentiality and anonymity of this research. All the self-administered questionnaires were in Chinese.

Finally, 1005 questionnaires were gathered in the period of 6 months, representing a 28.7% response rate. After removing missing or low-quality questionnaires, the final sample included 910 questionnaires. In final sample, 417 students were males (45.8%) and 493 students were females (54.2%). The average age of participants was 19.73 (SD = 1.365).

### 3.2. Measures

We list the questionnaire items in the Appendix A and describe the measures of the constructs as follows.

#### 3.2.1. Green Shared Vision

Green shared vision is measured by using a four-item scale developed by Chen et al. [4]. The seven-point Likert scale is used to measure a clear and ambitious environmental aspiration of the organization. A sample item is “A commonality of environmental goals exists in the university”. It has a Cronbach’s alpha of 0.85.

#### 3.2.2. Green Psychological Capital

We measured green psychological capital by referring to Chen and Yan [6]. A sample items is “Right now, I see myself as being pretty successful in green activities”. Responses are ranged from 1 (totally disagree) to 7 (totally agree). The twelve items have a Cronbach’s alpha of 0.92.

#### 3.2.3. Organizational Citizenship Behavior for the Environment

OCBE is captured by using a ten-item scale developed by Paillé et al. [60]. The seven-point Likert scale is used to measure Generation Z’s OCBE. A sample item is “I actively participate in environmental events organized by my university”. It has a Cronbach’s alpha of 0.89.

#### 3.2.4. Green Organizational Ambidexterity

An eight-item scale developed by Chen et al. [3] was used to measure green organizational ambidexterity. The seven-point measure captures the integration scientific ability of exploration and exploitation green capabilities. A sample item is “The university actively educates new green technology fields”. The Cronbach’s alpha of the eight items is 0.87.

#### 3.2.5. Environmental Attitude

An eight-item scale developed by Leonidou et al. [61] was used to measure environmental attitude. The seven-point Likert scale was applied to capture inward environmental attitude and outward environmental attitude. A sample item is “I am very concerned about the environment”. It has a Cronbach’s alpha of 0.88.

## 4. Results

### 4.1. Descriptive Statistics

Table 1 shows the means, standard deviations, correlation coefficients, and square root of a construct’s AVE. All the constructs in this study (GSV, GPC, OCBE, GOA, and EA) are significantly correlated with each other in Table 1.

### 4.2. Confirmatory Factor Analysis

Confirmatory factor analysis (CFA) is used to measure the goodness of model fit, and this study has excellent model fit results obtained for the five-factor model (χ^2^/df = 1.175, RMSEA = 0.014, CFI = 0.992, NFI = 0.946, TLI = 0.991, GFI = 0.954, AGFI = 0.949, SRMR = 0.024). If we do not consider the two moderators, green organizational ambidexterity and environmental attitude in the research model, the model fit results of the three-factor model (χ^2^/df = 1.217, RMSEA = 0.015, CFI = 0.994, NFI = 0.968, TLI = 0.994, GFI = 0.971, AGFI = 0.965, SRMR = 0.0203) are also acceptable.

### 4.3. Reliability and Validity

We tested the reliability and validity in several steps. Firstly, we assessed the reliability with Cronbach’s alpha, the results suggested that the Cronbach’s alpha of all the constructs (GSV, GPC, OCBE, GOA, and EA) are >0.7, consequently, the level of reliability is acceptable.

Secondly, all constructs have factor loadings greater than 0.5, the recommended threshold [62]. In addition, the values of composite reliability are greater than the suggested value of 0.70, and the values of average variance extracted (AVE) are also above the threshold of 0.50 [62], thus the level of convergent validity is acceptable.

Thirdly, discriminant validity is evaluated by comparing the square roots of AVEs with the corresponding correlations in Table 1, which reveals that the square roots of AVEs of all constructs are greater than the correlations between the constructs [63].

Based on the standardized factor loadings, CRs, and AVEs of the five-factor model presented in Table 2, there is satisfactory evidence for the reliability and validity in the five-factor model. We also demonstrated the standardized factor loadings, CRs, and AVEs of the three-factor model in Table 3, there is also satisfactory evidence for the reliability and validity the three-factor model.

### 4.4. Common Method Variance

The single source of data can result in the common method variance problem. We adopt the following approaches to avoid common method variance problem: First, we make sure the confidentiality and anonymity of the research in process control. Second, we try to make the questionnaire understandable and clear. Third, Harman’s one factor test is used to assess this problem and the result indicates that the variance explained by single factor is 31%.

### 4.5. Mediation Analysis

To explore the mediating effect of green psychological capital, we firstly check the direct effects between Generation Z’s green shared vision and OCBE. Table 4 shows that the total effect of Generation Z’s green shared vision on OCBE is significant [95% CI: (0.421, 0.573)]. After adding green psychological capital as a mediator, the direct effect of green shared vision on OCBE is significant [95% CI: (0.050, 0.210)]. At the same time, the indirect effect of green shared vision on OCBE is significant [95% CI: (0.299, 0.445)]. These results suggest that green psychological capital partially mediates the relationship between Generation Z’s green shared vision and OCBE; Hypothesis 1 (H_1_) is supported.

### 4.6. Moderation Analysis

**Figure 2:**
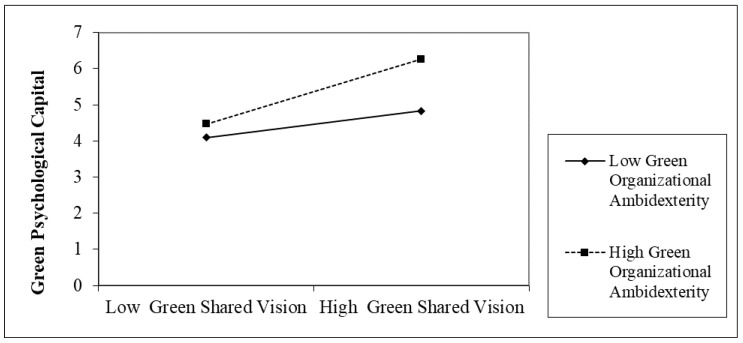
Moderating role of green organizational ambidexterity.

**Figure 3:**
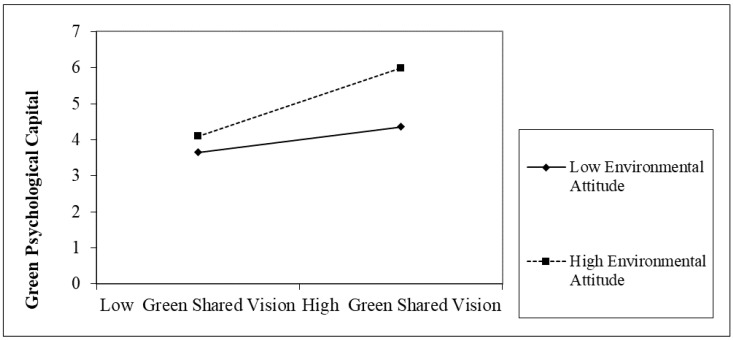
Moderating role of environmental attitude.

The results in Table 5 and Figure 2 indicate a significant coefficient interaction of green shared vision and green organizational ambidexterity (β = 0.262, *p* < 0.001). Moreover, the results in Table 6 and Figure 3 demonstrate a significant coefficient interaction of green shared vision and environmental attitude (β = 0.297, *p* < 0.001). Hypothesis 2a and 3a (H_2a_ and H_3a_) are such that the relationship between Generation Z’s green shared vision and green psychological capital is moderated by green organizational ambidexterity (GOA) and environmental attitude (EA).

### 4.7. Double Moderated Mediation Analysis

We applied SPSS PROCESS Model 9 [64] to test the moderated mediation effects. Table 7 reports the index of the moderated mediation effects and Table 8 shows the indirect effect of green shared vision–OCBE link at specific levels of green organizational ambidexterity (GOA) and environmental attitude (EA). The conditional indirect effect of Generation Z’s green shared vision on OCBE via green psychological capital moderated by green organizational ambidexterity is significant (index of moderated mediation = 0.0407, bootstrapping 95% CI [0.0182, 0.0624]). Moreover, the conditional indirect effect of Generation Z’s green shared vision on OCBE via green psychological capital is moderated by environmental attitude (index of moderated mediation = 0.0572, bootstrapping 95% CI [0.0367, 0.0779]. Table 7 indicates that the confidence intervals of the index of the moderated mediation do not contain zero, and thus H_2b_ and H_3b_ are supported.

The results in Table 8 demonstrate that boundary conditions of both green organizational ambidexterity (GOA) and environmental attitude (EA) exist in the green shared vision–green psychological capital–OCBE link. Interestingly, the indirect effects of Generation Z’s green shared vision on OCBE via green psychological capital is strongest when organizational ambidexterity (GOA) and environmental attitude (EA) are both at the highest level.

**Figure 4:**
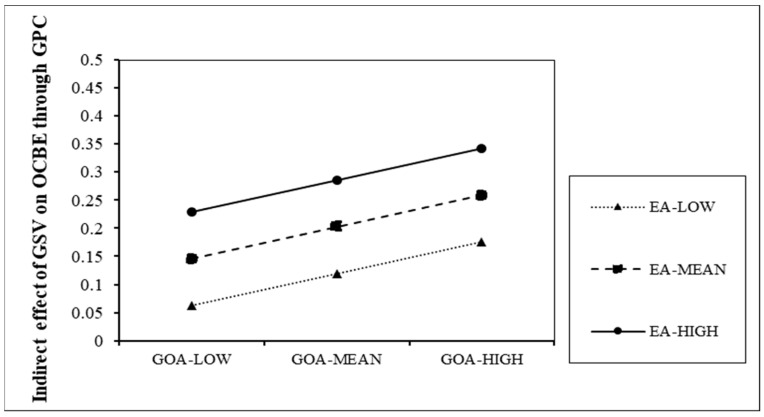
Indirect effect of green shared vision on OCBE via green psychological capital.

The moderated mediation exam is described by the Johnson–Neyman graph in Figure 4. The results indicate that a better green organizational ambidexterity and environmental attitude are both linked with a stronger indirect effect of Generation Z’s green shared vision on OCBE via green psychological capital.

## 5. Discussion

In this study, we address USR issues by explaining these two questions: (1) Does green psychological capital really act as a mediator in the green shared vision–OCBE link? (2) How would the interactions influence the relationship between green shared vision and OCBE via green psychological capital? Compared with traditional methods, we formed a double moderated mediation exam from 910 college students in southeast China. The main answers are listed as follows.

The positive psychology findings verify Hypothesis 1 (H_1_) which suggests that the green psychological capital performed a critical role between green shared vision and OCBE. This means that the green shared vision stimulates the Generation Z’s green psychological capital and in turn improves their OCBE. It also indicates the importance of green psychological capital among Generation Z in China. The antecedents and consequences of new notion “green psychological capital” and its specific four aspects “green self-efficacy”, “green optimism”, “green hope”, and “green resilience” have not been systematically explored. The results in this study extend the traditional research of psychological capital and perform a crucial guiding role in Generation Z’s future environmental actions.

In congruence of organizational ambidexterity, the findings verify Hypothesis 2 (H_2_) by demonstrating that green organizational ambidexterity is the boundary condition in the green shared vision–green psychological capital–OCBE link. This means universities with a higher capability of green organizational ambidexterity may have higher green shared vision, leading to stronger green psychological capital, thus promoting Generation Z to cope better with more unpredictable difficulties during environmental activities. The possible reason is that the higher capability of exploration and exploitation in environmental activities are both beneficial to the learning capabilities of college students, which might in turn increase Generation Z’s shared vision, creativity, and resilience in USR practices and improve their OCBE.

Consistent with social cognitive theory, the findings in this study verify Hypothesis 3 (H_3_) by indicating that environmental attitude also performs a moderated mediation role in the green shared vision–green psychological capital–OCBE link. In other words, Generation Z with better environmental attitude may have more green shared visions, leading to higher green psychological capital and stronger OCBE. Consistent with prior research [65,66], Generation Z’s environmental attitude is very important in USR education. Learning to care about environmental and social problems and accepting their social responsibilities is vital for young people.

In summary, we obtained adequate empirical support for our research framework by unpacking the black box of Generation Z’s green shared vision–OCBE link. In essence, we fill the research gap of USR by revealing the underlying mechanism and different interactions of this link, we also discuss the theoretical and practical implications of this study.

### 5.1. Theoretical Implications

Firstly, we lay the foundation for the development of theoretical integration linking four unique theoretical perspectives: social cognitive theory, positive psychology, broaden-and-build theory, and organizational ambidexterity. Although existing research focuses on some separate aspects or even a single theory in environmental literature, there is a research gap that does not fully capture the four theoretical perspectives in USR literature. To fill this gap, we offer an unstudied and cogent empirical framework linking green shared vision, green psychological capital, green organizational ambidexterity, environmental attitude, and OCBE. In doing so, we go beyond the existing limited theoretical perspectives and carry out a broader range of theoretical exploration.

Secondly, we further extend the growing psychological capital literature by incorporating it into environmental literature. We use “green psychological capital” and consider it as a bridging concept between Generation Z’s green shared vision and OCBE. We explore the underlying mechanism of green psychological capital theoretically and empirically. To be more specific, we provide newer and deeper insights into how green shared vision can improve Generation Z’s OCBE through green psychological capital, which is largely overlooked by existing research. This new construct explores an unstudied perspective of psychological capital in environmental protection and underscores the critical role of psychological capital in USR context.

Thirdly, we provide further insights and extend organizational ambidexterity theory. We contribute to organizational ambidexterity theory by firstly take the moderating role of green organizational ambidexterity into account. The results show that the indirect effect of Generation Z’s green shared vision on OCBE via green psychological capital is highest when green organizational ambidexterity is at the best level. In this way, we also extend the literature about green organizational ambidexterity [3]. The empirical results enhance extant research and reinforce the essential role of green organizational ambidexterity in sustainable literature.

### 5.2. Practical Implications

Firstly, we provide effective guidance and valuable details for green organizational ambidexterity, which should draw more public attention in USR practices. Resources and efforts should be provided to the integration of both exploratory and exploitative environmental capability in a creative way during USR practices, which might in turn increase Generation Z’s confidence in USR practices and improve their OCBE. As our results showed, green organizational ambidexterity performs a moderating role between Generation Z’s green shared vision and green psychological capital, and the indirect effects of Generation Z’s green shared vision on OCBE via green psychological capital is highest when green organizational ambidexterity (GOA) is at the strongest level. Consequently, this study shows that one way to enhance Generation Z’s OCBE is to take advantage of the green organizational ambidexterity (GOA) in universities, which can help them to overcome many difficulties in USR practices.

Secondly, we should not underestimate the vital role of the green psychological capital in USR practices. The findings in this study explore the underlying mechanism of green psychological capital theoretically and empirically. Moreover, our study also argues that universities should enhance Generation Z’s green psychological capital in USR practices, and they should inspire students by improving their “green optimism”, “green self-efficacy”, “green resilience”, and “green hope” in environmental activities.

Most important of all, our study offers real insight that USR practices form long-term and overall management including internal and external green activities. From the internal perspective, universities in China can carry out some frontier environmental research to improve their exploratory and exploitative environmental capabilities. At the same time, universities can provide more USR courses, thus Generation Z will establish the consciousness of energy-saving, food safety, waste prevention, etc. From the external perspective, universities in China should strengthen international exchange and cooperation with other universities as well as reinforce industry–university collaborations. The findings can broaden Generation Z’s green shared vision, which will motivate their OCBE and help Generation Z to perform their social responsibility.

## 6. Conclusions

To extend psychological capital literature, this study utilizes a new notion “green psychological capital” and explores an unstudied perspective of psychological capital in the field of environmental management. Future research can explore antecedents, consequences, or moderator roles of the green psychological capital based on the theoretical findings of our study. Moreover, future research can explore other interesting factors which can further extend our theoretical model. For example, this study identifies two constructs, green organizational ambidexterity and environmental attitude, which can moderate the green shared vision–OCBE link through green psychological capital. Future research can add new constructs, such as environmental culture, in the research model. By doing so, future research can extend the environmental literature in several ways and various details.

The data collected in this study are from Generation Z in Southeast China. As the development of environmental concept varies greatly among different regions in China, comparative studies of USR might be needed in different regions of China in future research. In addition, USR research can be further investigated in other developing countries with different populations. Therefore, the multi-country research with various economic and cultural environments might bring new insights of USR among Generation Z in the world.

Finally, as the development of USR is a dynamic process, another limitation of our study is the cross-sectional data. The longitudinal research can be conducted to find more details for Generation Z research. For example, researchers can take the COVID-19 into account, providing comparative studies of Generation Z’s OCBE pre-pandemic or post-pandemic. Moreover, researchers may also consider other long-term factors, such as international exchanges and cooperation between developing and developed countries. These suggestions can provide a comprehensive blueprint of USR research in the future.

## Figures and Tables

**Table 1:** Means, standard deviations, reliabilities, and correlations among study variables.

		Mean	S.D.	1	2	3	4	5	6	7
1	GENDER	0.46	0.50	-						
2	AGE	19.71	1.42	−0.03	-					
3	GSV	4.33	1.70	0.007	−0.056	(0.832)				
4	GPC	4.02	1.49	0.034	−0.051	0.526 ***	(0.724)			
5	OCBE	4.15	1.47	0.006	−0.049	0.473 ***	0.695 ***	(0.714)		
6	GOA	4.58	1.40	0.004	0.016	0.366 ***	0.408 ***	0.361 ***	(0.717)	
7	EA	4.61	1.45	−0.003	−0.018	0.290 ***	0.445 ***	0.419 ***	0.244 ***	(0.741)

^1^ *** *p* < 0.001 (two-tailed). ^2^ The data of the diagonal (in parentheses) are the square root of AVE (average variance extracted) of the construct. ^3^ GSV: green shared vision; GPC: green psychological capital; OCBE: organizational citizenship behavior for the environment; GOA: green organizational ambidexterity; EA: environmental attitude.

**Table 2:** Item loadings, Cronbach’s alpha, CR, and AVE of five factors.

	Factor Loadings
Green shared vision (GSV): α = 0.851, CR = 0.900, AVE = 0.692	
GSV1	0.832
GSV2	0.838
GSV3	0.822
GSV4	0.835
Green psychological capital (GPC): α = 0.917, CR = 0.929, AVE = 0.524	
GPC1	0.723
GPC2	0.685
GPC3	0.690
GPC4	0.737
GPC5	0.759
GPC6	0.740
GPC7	0.774
GPC8	0.753
GPC9	0.652
GPC10	0.735
GPC11	0.708
GPC12	0.723
Organizational citizenship behavior for the environment (OCBE): α = 0.893, CR = 0.912, AVE = 0.510	
OCBE1	0.700
OCBE2	0.702
OCBE3	0.715
OCBE4	0.708
OCBE5	0.712
OCBE6	0.736
OCBE7	0.725
OCBE8	0.679
OCBE9	0.728
OCBE10	0.735
Green organizational ambidexterity (GOA): α = 0.865, CR = 0.895, AVE = 0.515	
GOA1	0.726
GOA2	0.713
GOA3	0.744
GOA4	0.676
GOA5	0.737
GOA6	0.713
GOA7	0.709
GOA8	0.719
Environmental attitude (EA): α = 0.882, CR = 0.907, AVE = 0.548	
EA1	0.740
EA2	0.744
EA3	0.758
EA4	0.718
EA5	0.731
EA6	0.771
EA7	0.766
EA8	0.694

**Table 3:** Item loadings, Cronbach’s alpha, CR, and AVE of three factors.

	Factor Loadings
Green shared vision (GSV): α = 0.851, CR = 0.900, AVE = 0.692	
GSV1	0.832
GSV2	0.838
GSV3	0.822
GSV4	0.835
Green psychological capital (GPC): α = 0.917, CR = 0.929, AVE = 0.524	
GPC1	0.723
GPC2	0.685
GPC3	0.690
GPC4	0.737
GPC5	0.759
GPC6	0.740
GPC7	0.774
GPC8	0.753
GPC9	0.652
GPC10	0.735
GPC11	0.708
GPC12	0.723
Organizational citizenship behavior for the environment (OCBE): α = 0.893, CR = 0.912, AVE = 0.510	
OCBE1	0.700
OCBE2	0.702
OCBE3	0.715
OCBE4	0.708
OCBE5	0.712
OCBE6	0.736
OCBE7	0.725
OCBE8	0.679
OCBE9	0.728
OCBE10	0.735

**Table 4:** Mediational analysis (*n* = 910).

GSV—GPC—OCBE	Estimate	95% BC Bootstrapped CI [LL, UL]	Result
Total effects	0.493 ***	[0.421, 0.573]	significant
Direct Effects	0.124 **	[0.050, 0.210]	significant
Indirect Effect	0.368 ***	[0.299, 0.445]	significant

^1^ ** *p* < 0.01; *** *p* < 0.001 (two-tailed). ^2^ Lower and upper bound of 95% BC bootstrap confidence interval for that effect using 5000 bootstrap samples.

**Table 5:** Hierarchical regression results on green psychological capital moderated by green organizational ambidexterity.

	Green Psychological Capital			
	Model 1	Model 2	Model 3	Model 4
Step 1: Control variables				
Gender	0.097	0.089	0.087	0.098
Age	−0.052	−0.021	−0.031	−0.053
Step 2: Main effects				
Green shared vision (GSV)		0.780 ***	0.644 ***	0.633 ***
Step 3: Main effects				
Green organizational ambidexterity (GOA)			0.371 ***	0.452 ***
Step 4: Moderation effects				
GSV × GOA				0.262 ***
ΔR^2^	0.004	0.274	0.054	0.035
ΔF	1.648	344.185 ***	72.949 ***	50.458 ***
R^2^	0.004	0.278	0.332	0.367
Adj R^2^	0.001	0.276	0.329	0.364
Overall F	1.648	116.243 ***	112.343 ***	104.877 ***

^1^ *** *p* < 0.001 (two-tailed). ^2^ Green shared vision and green organizational ambidexterity are mean-centered for all analysis.

**Table 6:** Hierarchical regression results on green psychological capital moderated by environmental attitude.

	Green Psychological Capital			
	Model 1	Model 2	Model 3	Model 4
Step 1: Control variables				
Gender	0.097	0.089	0.093	0.098
Age	−0.052	−0.021	−0.021	−0.032
Step 2: Main effects				
Green shared vision (GSV)		0.780 ***	0.643 ***	0.651 ***
Step 3: Main effects				
Environmental attitude (EA)			0.475 ***	0.522 ***
Step 4: Moderation effects				
GSV × EA				0.297 ***
ΔR^2^	0.004	0.274	0.094	0.045
ΔF	1.648	344.185 ***	134.647 ***	69.751 ***
R^2^	0.004	0.278	0.371	0.416
Adj R^2^	0.001	0.276	0.369	0.413
Overall F	1.648	116.243 ***	133.704 ***	129.039 ***

^1^ *** *p* < 0.001 (two-tailed). ^2^ Green shared vision and environmental attitude are mean-centered for all analysis.

**Table 7:** Indices of moderated mediation.

Indices of Moderated Mediation				
GSV—GPC—OCBE	Green Organizational Ambidexterity			
	Index	BootSE	BootLLCI	BootULCI
	0.0407	0.0113	0.0182	0.0624
GSV—GPC—OCBE	Environmental Attitude			
	Index	BootSE	BootLLCI	BootULCI
	0.0572	0.0105	0.0367	0.0779

**Table 8:** Double conditional indirect effect of green shared vision–OCBE link at specific levels of green organizational ambidexterity (GOA) and environmental attitude (EA).

Variable				BC 5000 BOOT	
GOA	EA	IND	SE	LL95	UL95
**low**	**low**	0.0625	0.0217	0.0206	0.1053
**low**	**mean**	0.1456	0.0244	0.1005	0.1964
**low**	**high**	0.2288	0.0345	0.1638	0.2995
**mean**	**low**	0.1195	0.0205	0.0801	0.1612
**mean**	**mean**	0.2026	0.0192	0.1655	0.2406
**mean**	**high**	0.2857	0.028	0.2331	0.3418
**high**	**low**	0.1764	0.0295	0.1201	0.2373
**high**	**mean**	0.2595	0.0253	0.2111	0.3102
**high**	**high**	0.3427	0.0296	0.2852	0.4004

^1^ Coefficients represent specific indirect effects and standard errors at different values of both moderators, GOA and EA.

## Data Availability

The data is unavailable due to privacy.

## Appendix A. Questionnaire Items

**Constructs**	**Items**	**Content**
Green Shared Vision	GSV1	A commonality of environmental goals exists in the university.
	GSV2	A total agreement on the strategic environmental direction of the university.
	GSV3	All members in the university are committed to the environmental strategies.
	GSV4	All members of the university are enthusiastic about the collective environmental mission of the university.
Green Psychological Capital	GPC1	If I should find myself in a jam while engaging in green activities, I could think of many ways to get out of it.
	GPC2	Right now, I see myself as being pretty successful in green activities.
	GPC3	I can think of many ways to reach my current environmental goals.
	GPC4	I am looking forward to the green life ahead of me.
	GPC5	The future holds a lot of good in store for me in green activities.
	GPC6	Overall, I expect more good things to happen to me than bad in green activities.
	GPC7	Sometimes I make myself do green activities whether I want to or not.
	GPC8	When I’m in a difficult situation while engaging in green activities, I can usually find my way out of it.
	GPC9	It’s okay if there are people who don’t like me to participate in green activities.
	GPC10	I am confident that I could deal efficiently with unexpected environmental events.
	GPC11	I can solve most problems if I invest the necessary effort in green activities.
	GPC12	I can remain calm when facing difficulties in green activities because I can rely on my coping abilities.
Organizational Citizenship Behaviors for the Environment	OCBE1	In my work, I weigh my actions before doing something that could affect the environment.
	OCBE2	I voluntarily carry out environmental actions and initiatives in my daily activities at work.
	OCBE3	I make suggestions to my classmates about ways to more effectively protect the environment, even when it is not my direct responsibility.
	OCBE4	I actively participate in environmental events organized by my university.
	OCBE5	I stay informed about my university’s environmental initiatives.
	OCBE6	I undertake environmental actions that contribute positively to my organization‘s image.
	OCBE7	I volunteer for projects, endeavors or events that address environmental issues in my organization.
	OCBE8	I spontaneously give my time to help my classmates take the environment into account in everything they do at work.
	OCBE9	I encourage my classmates to adopt more environmentally conscious behavior.
	OCBE10	I encourage my classmates to express their ideas and opinions on environmental issues.
Green Organizational Ambidexterity	GOA1	The university actively introduces new generation of green products, services, or processes.
	GOA2	The university actively develops new green products, services, or processes.
	GOA3	The university actively finds new green markets.
	GOA4	The university actively educates new green technology fields.
	GOA5	The university actively improves existing green products, services, or processes.
	GOA6	The university actively adjusts existing green products, services, or processes.
	GOA7	The university actively consolidates existing green markets.
	GOA8	The university actively reinforces existing green technology fields.
Environmental Attitude	EA1	I am very concerned about the environment.
	EA2	I would be willing to reduce my consumption to help protect the environment.
	EA3	I would give part of my own money to help protect wild animals.
	EA4	I have asked my family to recycle some of the things we use.
	EA5	Major political change is necessary to protect the natural environment.
	EA6	Anti-pollution laws should be enforced more strongly.
	EA7	Major social changes are necessary to protect the natural environment.
	EA8	Humans are severely abusing the environment.
